# Unusual Dual Brain Tumor Morphologies in an MEN1 Patient: A Case Report of Diagnostic Challenges and Methylation Insights

**DOI:** 10.3390/ijms262010065

**Published:** 2025-10-16

**Authors:** Viharkumar Patel, Orwa Aboud, Abdelrahman Barakat

**Affiliations:** 1 Department of Pathology, University of California Davis Health System, Sacramento, CA 95817, USA; vppatel@health.ucdavis.edu; 2 Department of Neurology and Neurological Surgery, University of California Davis Health System, Sacramento, CA 95817, USA; 3 Department of Pathology, University of Iowa, Iowa City, IA 52242, USA

**Keywords:** multiple endocrine neoplasia type 1, typical teratoid/rhabdoid tumor, primitive neuroectodermal tumors, hematoxylin and eosin, pituitary adenoma, methylation, next generation sequencing

## Abstract

Multiple Endocrine Neoplasia Type 1 (*MEN1*) is an autosomal dominant disorder commonly associated with tumor development in the parathyroid glands, pancreas, and pituitary gland. While pituitary adenomas are frequently observed in *MEN1* patients, the presence of additional tumors within the pituitary gland is unusual. Moreover, the co-occurrence of a pituitary adenoma with an atypical teratoid/rhabdoid tumor (ATRT) has not been previously documented. ATRT is a rare, aggressive neoplasm predominantly affecting young children and is typically associated with inactivating mutations in the *SMARCB1* or *SMARCA4* tumor suppressor genes. These mutations result in uncontrolled cellular proliferation, which underlies the malignancy’s rapid progression. In adults, ATRT is exceedingly rare, making this case particularly noteworthy for its uniqueness in both tumor type and patient demographics. ATRTs are now classified into three molecular subgroups—*MYC*, *SHH*, and *TYR*—each with distinct epigenetic and clinical features, further refining diagnostic and prognostic assessments. In this case report, we describe a case of a female patient with *MEN1* who experienced several recurrences of pituitary adenoma, ultimately necessitating surgical resection. Detailed pathological evaluation of the resected tissue revealed two distinct neoplasms within the pituitary gland: one typical of a pituitary adenoma, and the other confirmed as ATRT. The diagnosis of ATRT was established through extensive workup including immunohistochemical analysis, next-generation sequencing and methylation profiling, which served as essential tools in distinguishing ATRT from other potential differential diagnoses. This case illustrates the complex diagnostic journey and challenges encountered in identifying ATRT in the context of *MEN1*, underscoring the importance of using advanced molecular and immunohistochemical techniques in atypical presentations. Furthermore, it expands the understanding of potential tumor associations within *MEN1*, providing insight for pathologists and clinicians into the rare possibility of concurrent tumors in addition to pituitary adenoma in *MEN1* patients. Raising awareness of such co-occurrences could prompt earlier diagnostic considerations by refining the differential diagnosis in patients with *MEN1* presenting with unusual tumor types.

## 1. Introduction

Multiple Endocrine Neoplasia type 1 (*MEN1*) is a hereditary syndrome characterized by the development of tumors in multiple endocrine glands. The most common manifestations of *MEN1* include parathyroid, pancreatic, and pituitary tumors [1]. These tumors are often the first indication of *MEN1* and lead to a variety of clinical symptoms depending on the hormones involved and the size of the tumors [2].

Pituitary adenomas, one of the hallmark tumors of *MEN1*, arise from the anterior pituitary gland and are usually benign. These adenomas can secrete excess hormones, leading to conditions such as hyperprolactinemia, acromegaly, or Cushing’s disease, depending on the type of hormone produced [3]. In *MEN1* patients, pituitary adenoma is a common pathology occurring in approximately 30–40% of the cases [3,4].

Many case reports have documented the co-occurrence of another tumor with a pituitary adenoma in *MEN1* syndrome [5], and this case contributes to this body of evidence. Atypical teratoid/rhabdoid tumor (ATRT) is a rare and highly aggressive tumor primarily occurring in the central nervous system of young children. The occurrence of ATRT in adults is rare, and the co-occurrence of ATRT in an adult patient with *MEN1* has not been reported. In adult females, ATRTs have been found in the sellar region. ATRTs are challenging to diagnose due to their rarity and in this case, its presentation in a syndrome. This may necessitate advanced molecular techniques, such as methylation profiling, to distinguish them from other tumors more commonly seen in *MEN1*, such as meningioma. ATRTs often exhibit deletions or mutations in the *SMARCB1* gene, which is commonly associated with this tumor type [6].

In this case report, we present a unique case of a patient with *MEN1* syndrome who developed two distinct tumors in the brain: a typical pituitary adenoma and an ATRT. This dual pathology highlights the diagnostic challenges and the importance of considering a broad differential diagnosis in patients with *MEN1*, especially when encountering unusual tumor morphologies.

## 2. Detailed Case Description

A 38-year-old female with a history of multiple endocrine neoplasia type 1 (*MEN1*) and macroprolactinoma initially presented with amenorrhea in 2004, leading to the discovery and subsequent resection of her prolactinoma. Notably, in November 2003, she underwent a three-gland parathyroidectomy to address her hyperparathyroidism, reflecting the systemic nature of her endocrine disease. Since that time, she was lost to follow-up and no clinical, radiologic, or pathologic information was obtained.

**Figure 1:**
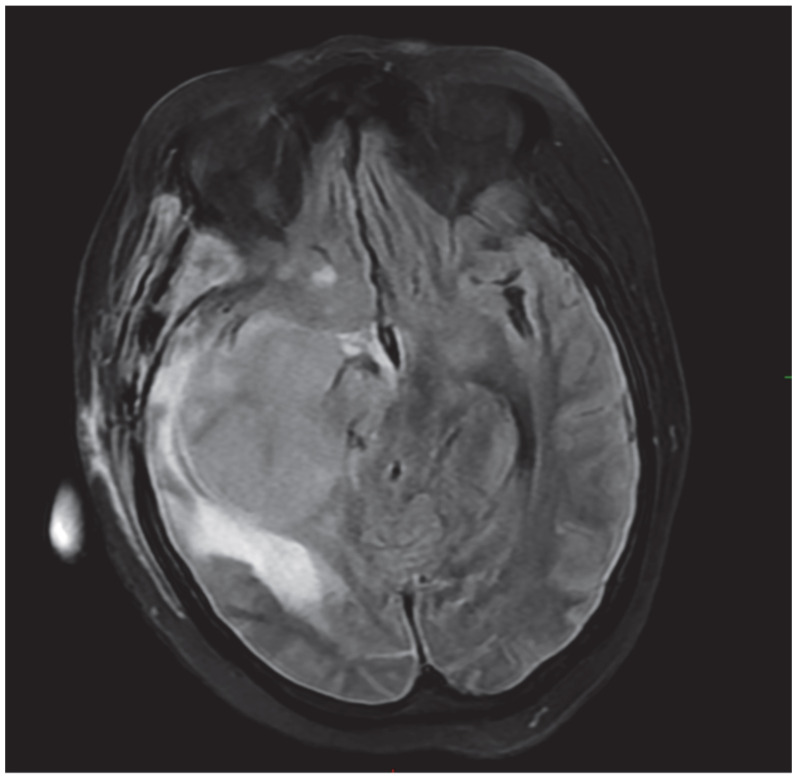
Axial T2 FLAIR showing large right intra-axial temporal lobe enhancing mass with surrounding vasogenic edema and mass effect.

Her medical journey took a concerning turn in 2013 when she returned with a significantly enlarged pituitary macroadenoma, with extensive invasion into the right subfrontal region, right middle cranial fossa, posterior cranial fossa, and bilateral cavernous sinuses (Figure 1). Despite a subtotal resection, radiation therapy, and additional medication, the full scope of her treatment and follow-up was compromised by repeated loss to follow-up and inconsistent medication adherence.

Over the years, her engagement with healthcare services waxed and waned until a severe incident in 2024. She experienced a blackout while driving, leading to a motor vehicle accident. Subsequent medical evaluations highlighted new health complaints, including significant weight gain, shortness of breath, and mobility issues that severely limited her ability to work. She also suffered from episodes of “spacing out,” left-sided headaches, and vomiting.

In the wake of these symptoms, imaging studies revealed a significant mass in the sellar and suprasellar regions extending into the adjacent right middle cranial fossa and the medial temporal lobe. This mass was associated with large volume intraparenchymal and intraventricular hemorrhage, hydrocephalus, and blood products scattered across various intracranial sites. These findings raised concerns about the recurrence of her adenoma with progression to pituitary carcinoma.

**Figure 2:**
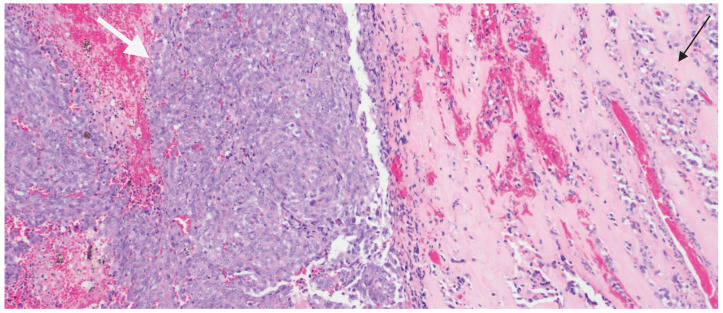
H&E staining shows two distinct tumor morphologies. The highly cellular sheets of malignant tumor cells is highlighted by the white arrow, while the nests of pituitary adenoma cells is highlighted by the black arrow. The pink acellular stroma in between the adenoma cells is treatment effect (10×).

During the surgical intervention in 2024, what was assumed to be a single tumor turned out to be two distinct tumor entities histopathologically, with two different adjacent tumor morphologies (Figure 2). One tumor was a pituitary adenoma with extensive treatment effect. The adenoma cells formed small compact nests and were surrounded by abundant hyalinized stroma, supported by immunohistochemistry and special stain studies. The other tumor differed markedly in both morphology and immunohistochemistry. The second tumor was hypercellular, consisting of sheets of tumor cells that often-exhibited spindle-cell and focally rhabdoid morphology. These tumor cells had a distinct immunophenotype not consistent with a neuroendocrine tumor. This discrepancy led to a comprehensive diagnostic approach, including various immunohistochemical stains to exclude possible diagnoses in a wide differential such as meningioma, metastatic melanoma, carcinoma, and pituitary/neuroendocrine neoplasms; however, the results were inconclusive.

Therefore, the tissue was subjected to molecular and methylation profiling to verify its nature. The results of molecular testing were consistent with a diagnosis of an atypical teratoid/rhabdoid tumor. Sequencing revealed a *MEN1* somatic alteration (*MEN1* L39FS*77) in the ATRT component, reflecting the presence of *MEN1*. Notably, possible radiation-induced neoplasia was further excluded by the lack of a high mutation burden rate via Foundation One testing.

This case report aims to raise awareness of the non-classical pathologies that might be encountered in patients with multiple endocrine neoplasia type 1 (*MEN1*) and to highlight the utility of molecular testing to achieve the correct diagnosis. Such findings underscore the necessity for a broad diagnostic lens and the importance of considering a wide range of differential diagnoses when dealing with brain tumors in *MEN1* patients, especially when the clinical presentation raises concerns about disease progression.

## 3. Discussion

Multiple endocrine neoplasia type 1 (*MEN1*) is an autosomal dominant disorder characterized by the occurrence of tumors in multiple endocrine glands, primarily affecting the parathyroid glands, anterior pituitary, and pancreatic islets. It is a rare autosomal dominant disorder, resulting from pathogenic inactivating variants in the *MEN1* gene, a tumor suppressor located on chromosome 11q13 [2]. Classically, *MEN1* is associated with primary hyperparathyroidism, which occurs in over 90% of cases, pituitary adenomas, and neuroendocrine tumors of the gastrointestinal tract, particularly in the pancreas. Patients with *MEN1* frequently develop cutaneous tumors, including lipomas, collagenomas, and fibrovascular facial tumors [2,5,7,8].

Pituitary tumors in *MEN1* cases show diverse hormonal activity: 60% secrete prolactin, around 25% secrete growth hormone, and 5% secrete corticotropin. This can result in clinical manifestations such as hyperprolactinemia (including amenorrhea, galactorrhea, and infertility), acromegaly, or Cushing’s disease. Furthermore, these tumors can compress surrounding tissues, potentially causing visual problems, such as bitemporal hemianopsia or hypopituitarism [4,7]. In addition to pituitary tumors, the syndrome has also been reported to be linked to non-endocrine brain tumors such as meningiomas and xanthoastrocytomas. However, none of these reported cases included ATRTs [4,7,8,9].

Sporadic ATRTs are notable for their aggressive behavior and poor prognosis, particularly associated with mutations in the *SMARCB1* or *SMARCA4* tumor suppressor genes [6,10], leading to unchecked cellular proliferation and tumor development [11,12]. The typical presentation of ATRTs includes rapid onset of neurological symptoms such as headaches, seizures, and significant changes in motor skills or alertness, reflecting their fast-growing nature and critical locations within the CNS [10,13,14].

Atypical teratoid/rhabdoid tumors (ATRTs) represent a small fraction, about 1–2%, of all pediatric brain tumors, making them quite rare. They predominantly affect very young children, typically under the age of 3, and it is uncommon to see these tumors in children older than 6 years [14]. The most common location of occurrence of ATRT in pediatric patients is within the posterior fossa of the brain. As they often present early in life, the differential diagnoses include other malignant and aggressive tumors such as medulloblastoma, pediatric high-grade glioma, and even anaplastic ependymoma. ATRTs are even rarer in adults, underscoring their primary association with early childhood [15].

ATRTs are also seen in genetically inherited tumor predisposition syndromes, most notably rhabdoid tumor predisposition syndrome (RTPS) [10]. RTPS is caused by germline mutations in *SMARCB1* and/or *SMARCA4* along with somatic mutations in the remaining copy of *SMARCB1* (RTPS1) and/or *SMARCA4* (RTPS2). Clinical manifestations present early on in childhood characterized by multiple aggressive rhabdoid tumors occurring at virtually any anatomic site; the cerebellum is the most common location in the brain. Extracranial rhabdoid tumors are seen in the head and neck, intra-abdominal organs, retroperitoneum, paravertebral skeletal muscle, the heart, and kidney (also known as rhabdoid tumor of kidney).

Grossly, these rhabdoid tumors can appear as large, bulky masses that are usually located supratentorially in adults [10]. Microscopically, ATRTs are characterized by the presence of rhabdoid cells, which are large cells with eosinophilic cytoplasm, globular cytoplasmic inclusions and prominent nucleoli. The tumors may also contain small and large cells with variable morphologies, including epithelioid and spindle-shaped cells, and may exhibit areas resembling primitive neuroectodermal tumors (PNETs) [10,16,17]. The varying morphologies of the tumor cells raise a broad differential as many malignant tumors of diverse differentiation, such as carcinoma, melanoma, and sarcoma can show these morphologic features. Without immunohistochemical studies and often molecular analyses, the diagnosis would remain elusive. Furthermore, it is unusual that a relatively simple recurrent molecular alteration, such as bi-allelic inactivation of *SMARCB1* produces a tumor with quite diverse morphologic features and clinical heterogeneity.

Indeed, recent research using DNA methylation analyses and gene expression profiling has identified three distinct molecular subtypes that could potentially explain the different observed morphologies seen with ATRT: ATRT-*TYR* (*Tyr*osinase, MiTF, BAMP4 overexpression), ATRT-*SHH* (Sonic hedgehog), and ATRT-*MYC* (*MYC* overexpression without germline amplification) [17,18]. There are clinical differences in these three major subgroups of ATRT. The ATRT-*TYR* subgroup is the youngest group, often presenting within the first two years of life, mostly involves the posterior fossa, shows inactivating *SMARCB1* mutation along with loss of chromosome 22q and shows global DNA hypermethylation. The ATRT-*SHH* subgroup is seen in supra- and infratentorial locations, has compound heterozygous *SMARCB1* point mutations, and may have better prognosis with *ASCL1* (Notch pathway) mutations. The ATRT-*MYC* subgroup has the highest median age group of 27 months in pediatric populations and is the most common adult ATRT subtype; 50% of these are supratentorial and show *SMARCB1* homozygous deletions [17].

In this case, at the time of frozen section, the expectation was that of a pituitary adenoma. Typically, adenoma tumor cells smear very well when performing the intraoperative squash preparation. When viewing the smear under the microscope, one sees a sea of small-to-medium-sized round nuclei with stippled chromatin, consistent with the neuroendocrine features of an adenoma. The subsequent frozen section is corroborative. However, during the intraoperative consultation, the smear and frozen section revealed a sheet-like growth pattern formed by epithelioid to rhabdoid-appearing cells with prominent nucleoli, densely eosinophilic cytoplasm and atypical mitotic figures. Atypical mitotic figures are a hallmark of malignancy. Furthermore, the varied morphologic features were not consistent with pituitary adenoma, and concern was raised for a malignant neoplasm.

**Figure 3:**
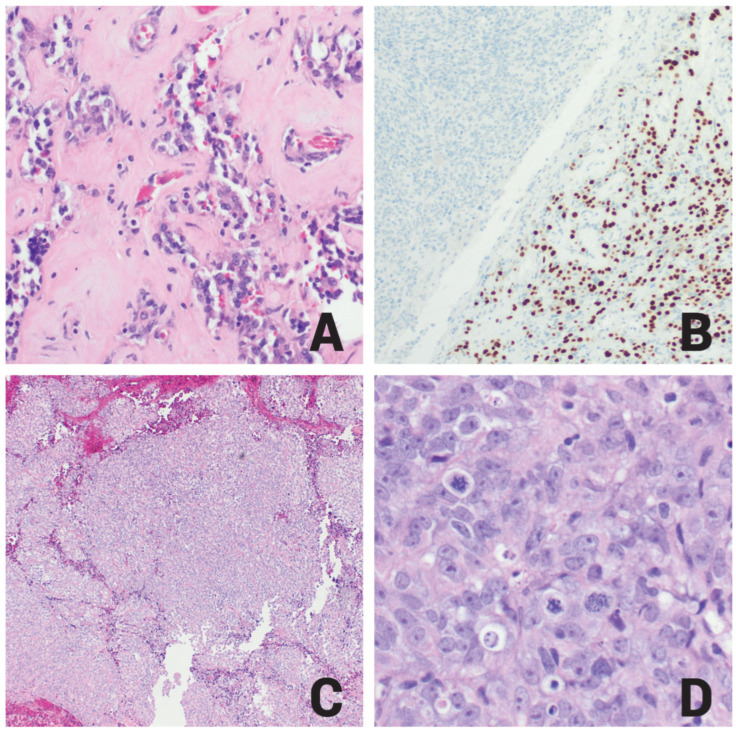
(**A**) H&E staining reveals a pituitary adenoma with glandular formation, composed of neuroendocrine cells displaying uniform nuclei, stippled chromatin, inconspicuous nucleoli, and moderate cytoplasm (20×). (**B**) PIT1 immunohistochemistry demonstrates positive nuclear staining in the pituitary adenoma (right side) but is negative in the ATRT (left side) (20×). (**C**) H&E staining of ATRT shows tumor cells arranged in a sheet-like pattern (10×). (**D**) H&E staining of ATRT highlights epithelioid to rhabdoid-appearing cells with prominent nucleoli, densely eosinophilic cytoplasm, and atypical mitotic figures (40×).

Examination of the formalin-fixed paraffin-embedded tissue revealed two distinct histopathologic tumors. The first showed typical morphology of glandular architecture of neuroendocrine cells having uniform nuclei with stippled chromatin, inconspicuous nucleoli, and moderate amount of cytoplasm (Figure 3A), consistent with the pituitary adenoma component. The tumor cells formed small, compact acinar structures embedded in an abundantly eosinophilic acellular stroma. This characteristic appearance is seen in pituitary tumors with treatment effect. Reticulin special stain showed loss of reticulin fibers in the adenoma component. The diagnosis of pituitary adenoma was further supported by a positive staining reaction for PIT1 (Figure 3B) and prolactin, consistent with the patient’s known pituitary adenoma.

**Figure 4:**
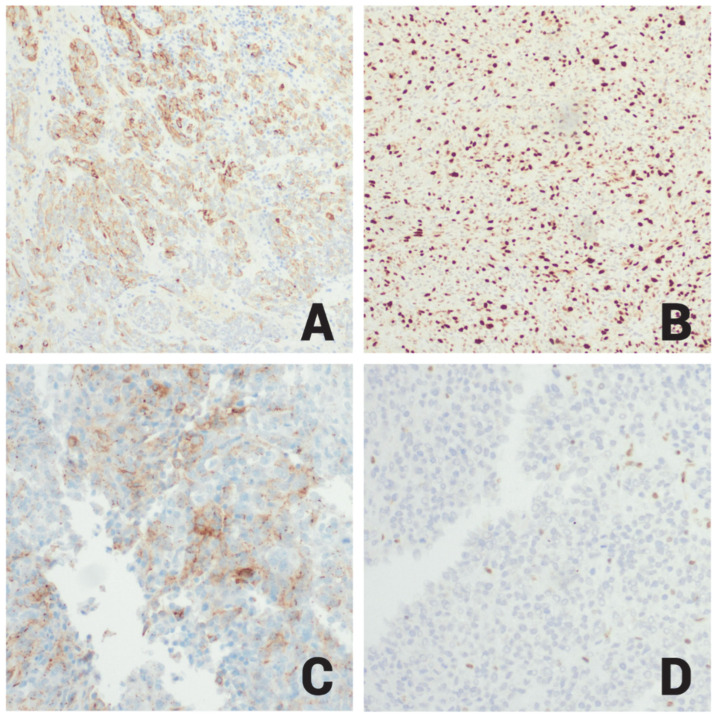
(**A**) Immunohistochemistry for EMA showing patchy membranous staining reaction in ATRT (20×). (**B**) Immunohistochemistry for Ki67 in ATRT showing high proliferative index activity exhibited by a positive nuclear staining reaction in about 60% of the tumor cells (20×). (**C**) Immunohistochemistry for SSTR2 in ATRT showing focal membranous staining reaction (20×). (**D**) Immunohistochemistry for INI1 showing negative staining reaction in ATRT cells (20×).

The second component showed sheets of rhabdoid (Figure 3C) and spindle cells with high mitotic activity, atypical mitoses (Figure 3D), areas of necrosis, and infiltration of non-neoplastic cerebral cortex brain tissue. Immunohistochemically, this component showed negative staining reactions for CK7, CK20, CK5/6, P40, D2-40, P63, BAP1, desmin, myogenin, STAT6, ER, PR, SOX10, S100, MiTF, Melan A, HMB45, BRAF V600E, synaptophysin, INSM1, PIT1 (Figure 3B), prolactin, TPIT, SF-1, LH, FSH, TSH, GH, and ACTH. It also showed focal positive staining reactions for AE1/AE3, CAM5.2, H3K27ME3, EMA (Figure 4A), P53 (20%), a high Ki67 proliferative index (60%) (Figure 4B), and patchy SSTR2 (Figure 4C) positivity; E-cadherin was retained, and reticulin staining highlighted reticulin investment of the neoplastic cells (Table 1).

With respect to the immune profile and histologic pattern, all the possible differential diagnoses were excluded except for anaplastic meningioma such as rhabdoid meningioma, a CNS WHO grade 3 malignant tumors. Although meningiomas are not commonly associated with *MEN1* syndrome, there have been reports of meningiomas in families with *MEN1*. While SSTR2 and EMA were focally positive, the intensity and patchy nature of the staining were not completely convincing. Furthermore, BAP1 an immunohistochemical marker that is lost in rhabdoid meningiomas, was retained in this case, excluding rhabdoid meningioma as a diagnosis. Consequently, tissue samples containing both the pituitary adenoma component and the rhabdoid tumor component in the same slide were sent for next generation sequencing, revealing a *MEN1* p. L39fs frameshift mutation, an *MRE11* splice site mutation, homozygous *CDKN2A*/*B* deletion, and loss of proximal 22q. The *MEN1* p. L39fs and *MRE11* mutations are recurrent mutations consistently seen in *MEN1* syndrome and represent the pituitary adenoma component. *CDKN2A*/*2B* homozygous loss is seen in several malignant and biologically aggressive tumors, including high-risk neuroendocrine pituitary tumors. Interestingly, no *SMARCB1* or *SMARCA4* mutations were detected from NGS studies. However, losses of proximal 22q were seen. At this time, the diagnosis of recurrent pituitary adenoma was confirmed, but it was unclear if the second tumor was de novo synchronous malignancy, or a *MEN1*-driven non-pituitary malignant neoplasm. In addition, large scale chromosomal alterations were seen that included gains of 1q and 14q, and losses of proximal 15q in addition to proximal 22q. To investigate further, a different block of tissue, that consisted solely of the rhabdoid tumor morphology was sent for DNA methylation profiling. This revealed a high-confidence match to ATRT and showed loss of *SMARCB1* on copy number analysis. Subsequently INI-1 (*SMARCB1*) immunohistochemistry was performed showing loss of INI-1 expression (Figure 4D), consistent with ATRT. From a clinical perspective, this ATRT most likely fits into the ATRT-*MYC* subgroup given its supratentorial location in an adult individual. The DNA methylation profile did not subclassify the subgroup of ATRT.

It is important to note that classically, ATRT presents in childhood, but there are now known to be three subgroups of ATRT that represent clinical and histologic heterogeneity. A prime question in this case was whether the ATRT, once discovered, was due to *MEN1* syndrome, a sporadic, secondary malignancy, or radiation-induced secondary malignancy. No molecular evidence suggesting radiation-induced injury was seen, given a low tumor mutation burden. Even in the initial NGS study that revealed the *MEN1* alterations, there was a hint of the presence of ATRT through the combination of *CDKN2A*/*B* homozygous loss and proximal 22q loss, but the additional chromosomal alterations masked this revelation. Once the DNA methylation analysis on the rhabdoid tumor sample revealed a high-confidence match to ATRT and loss of *SMARCB1*, the diagnosis of ATRT was confirmed with loss of INI-1 expression in the rhabdoid tumor component.

## 4. Conclusions

In conclusion, our case represents a rare occurrence of ATRT in a patient with MEN-1 syndrome. This is notable both due to the presentation of ATRT in an adult, its occurrence in a patient with MEN-1 syndrome, and the advanced molecular and immunohistochemical testing to confirm the diagnosis of two synchronous but separate tumors.

## Figures and Tables

**Table 1:** Summary of immunohistochemical and special staining in workup of pituitary adenoma and ATRT.

Immunohistochemical Workup	Result	Notes
CK7, CK20, CK5/6, P40, D2-40, P63, BAP1, Desmin, Myogenin, STAT6, ER, PR, SOX10, S100, MiTF, Melan A, HMB45	Negative	—
BRAF V600E, Synaptophysin, INSM1, TPIT, SF-1, LH, FSH, TSH, GH, ACTH	Negative	
AE1/AE3, CAM5.2, H3K27ME3	Negative	
PIT1, Prolactin	Positive	PIT1 shown in Figure 3B
EMA	Focal Positive	EMA shown in Figure 4A
P53	Positive (20%)	—
Ki-67	High (60%)	High proliferative index (Figure 4B)
SSTR2	Patchy Positive	Shown in Figure 4C
INI-1	Positive	Loss of nuclear INI1 expression in ATRT cells (mutant); Figure 4D
E-cadherin	Retained	—
Reticulin stain	Positive	Highlights loss of reticulin investment in adenoma cells;

## Data Availability

The original contributions presented in this study are included in the article. Further inquiries can be directed to the corresponding author.
